# With Appreciation

**DOI:** 10.1002/jcsm.13686

**Published:** 2024-12-11

We thank the following individuals who served as manuscript reviewers for The Journal of Cachexia, Sarcopenia and Muscle (JCSM) in 2024.

We highly appreciate their efforts.

Fair, conscientious and timely peer reviews contribute to the success of JCSM.

The Editors.

## 6 and more reviews

Olasunkanmi Adegoke

Jiyun Ahn

Amy Attaway

Vickie Baracos

Patricia Batista

Catherine Bellissimo

Philip Bonomi

Leo Brown

Liang‐Kung Chen

Xian Wu Cheng

Wai Cheung

Jeffrey Crawford

Rafaela Espírito Santo

Jens Fielitz

Denis Guttridge

Yoshitaka Hashimoto

Satoshi Ikeda

Hee‐Won Jung

Serkan Kir

Masaaki Konishi

Barry Laird

Ning Li

Ryan McGrath

Tateaki Naito

Gopal Nambi

Miguel Naveira

Amirabbas Nikkhah

Mats Nilsson

Masatsugu Okamura

Luciana Pereira

Ishan Roy

Guo‐Tian Ruan

Kunihiro Sakuma

Hiroshi Shamoto

Mitsuhiro Shimura

Liz Simon Peter

Vanessa Stadlbauer

Ming Yang

Yoshihiro Yoshimura

## 4–5 reviews

Belgin Akan

Eva Alves

Elia Angelino

Nile Banks

Jing‐Fu Bao

Jose Barreto Carvalheira

Linda Beenet

Laure B. Bindels

Richard Bohannon

Miguel Borda

Arben Boshnjaku

Marina Bouche

Jeffrey Brault

Danna Breen

Dean Campelj

An‐Chih Chang

Wei Chen

Zi Chen

Paola Costelli

Brittany Counts

Adeline Dolly

Richard Ehman

Mert Eşme

Susanne Fleig

Wie Gao

Nicholas Greene

Daniel Ham

Tewfik Hamidi

Sabah Hussain

Krystian Josiak

Nicole Kiss

Adam Kuchnia

Wei‐Ju Lee

Daniel Marks

Radmila Matijevic

Alexandra Mayhew

Chris McGlory

Benjamin Miller

Yusuke Nishimura

Mario Perez‐Zepeda

Christopher Perry

Carla Prado

Andrea David Re Cecconi

Vanina Romanello

Megan Rosa‐Caldwell

Akihiro Sakuyama

Rengfei Shi

Ng Shyh‐Chang

Brandon VanderVeen

Noah Weisleder

Leo Westbury

Sandra Zampieri

Silke Zimmermann

## 2–3 reviews

Ece Acar

Hiroaki Adachi

Volker Adams

Bumsoo Ahn

Takeru Akabane

Naoki Akazawa

Julian Alcazar

Matthew Alexander

Tiago Alexandre

Hazim Alhiti

Hamed Alizadeh pahlavani

Stephen Alway

Hilman Amin

Ethan Anderson

Hidenori Arai

Amelia Aranega

Gopalakrishnan Aneeshkumar Arimbasseri

Paige Arneson‐Wissink

Christopher Axelrod

Martin Bahls

Luke Baker

Omar Baritello

Elisabeth Barton

Joshua Barzilay

Yuri Battaglia

Philipp Baumert

Annette Bellar

Marc Beltrà

Maximilien Bencze

Cécile Bétry

Jose‐Ramon Blanco

Marc Bonnefoy

Piers Boshier

Pedro Braga

Ellen Breen

David Brigstock

Marco Brotto

Jacob Brown

Henver Brunetta

Stefan Buettner

Daniel Bunout

Barrington Burnett

Xiaoling Cai

Anlan Cao

Luca Caputo

Pierre Carlier

Luka Cavka

Joe Chakkalakal

Antoine Chatrenet

Gang Chen

Wing Hoi Cheung

Shotaro Chubachi

David Church

Aleksandar Cirovic

Stephanie Compton

Maria Conte

Javier Courel‐Ibáñez

David Currow

Aneesha Dasgupta

Boel De Paepe

Anton De Spiegeleer

Rafael Deminice

Fabio Demontis

Ross Dolan

Stefano Donega

Michael Drey

Randall D'Souza

Stephanie Duguez

Delphine Duteil

Peter Edholm

Reilly Enos

William Evans

Nir Eynon

Sestina Falcone

Alessandro Fanzani

Ellis Fok

Guilherme Fonseca

Armin Frille

Qi Fu

Michael Fuchs

Tania Garfias‐Veitl

Karim Gariani

Se‐Il Go

Lijing Gong

Gilles Gouspillou

Pol Grootswagers

Jenny Gunton

Baosheng Guo

Mark Hamrick

Lauren Hanna

Henner Hanssen

Koichiro Hayashi

Russell Hepple

Megan Herodes

Steven Heymsfield

Michael Hicks

Chris Hine

Eric Hoffman

Yan Huang

Chun‐Feng Huang

David Hughes

Joshua Huot

Chikwendu Ibebunjo

Hikaru Ihira

Carl‐Philipp Jansen

Honglei Ji

Zhenzhou Jiang

Young Su Joo

Sarah Judge

Kuniyasu Kamiya

Haiming Kerr

Teng Keen Khong

Sunyoung Kim

Beom‐Jun Kim

Su Jin Kim

Ben Kirk

Joost Klaase

Frederick Koh

Alexander Krannich

Martin Krssak

Ashok Kumar

Karsten Kummer

Kenjiro Kunieda

Atsushi Kuno

Soon Hyo Kwon

Mitja Lainscak

Laurence Lapauw

Jean‐Philippe Leduc‐Gaudet

Ji Hyun Lee

Jie Lee

Mingxing Lei

Yi‐Ping Li

Shengxu Li

Lei Li

Vitor Lira

Ya‐Wen Liu

Kenneth Lo

Goran Loncar

Felipe V C Machado

Keisuke Maeda

Joris Mallard

Giovanni Mantovani

James Markworth

Lisa Martin

Brenda‐Eugenia Martinez‐Herrera

Stephan Matecki

Hiromi Matsumoto

Alvin Matsumoto

Hamish McAuley

Donald McMillan

Christopher Mejias

Cláudia Mendes

Manuela Merli

Graziella Messina

Masaki Mogi

Ryan Montalvo

Elena Monti

Aaron Morton

Nobuto Nakanishi

Hiroshi Nishimune

John Noone

Kristina Norman

Masafumi Nozoe

Everson Nunes

Kirsten Nyrop

Takuro Okamura

Masatsugu Okamura

Brian O'Neill

Theng Choon Ooi

Cameron Oswalt

Mati Pääsuke

Allan F. Pagano

Giuseppe Paolisso

Myung Woo Park

Evasio Pasini

Tao‐Chun Peng

Fabio Penna

Leonardo Pereira

Éder Petry

Iain Phillips

Felix Piecha

Mindy Pike

Ariane Pille

Sara Pilotto

Addolorata Pisconti

Marco Ponzetti

Michael Praktiknjo

S Russ Price

Oliver Price

Melissa Puppa

Pier Lorenzo Puri

Joost Raaphorst

Jérémy Raffin

Md Mamunur Rashid

Clara Rentz

Charles Rice

Paul Roberson

Luise Roehrich

Leonardo Roever

Maria Rohm

Daniel Runco

Joseph Rupert

Terence Ryan

Nizar Saad

Kotomi Sakai

Sadhana Samant

Konda Mani Saravanan

Takayoshi Sasako

Shuichi Sato

Isabelle Savary‐Auzeloux

Rebecca Schmitt

Stylianos Scordilis

Idowu Senbanjo

Guo‐kai Shang

Takehito Shukuya

Nico Sollmann

Thomas Stehlé

Jennifer Steiner

Klaus Steinmeyer

Barbara Strasser

Paweena Susantitaphong

Norio Suzuki

Yasuharu Tabara

Erin Talbert

Avesh Thuluvath

Malte Tiburcy

Paul Titchenell

Caterina Trevisan

Capucine Trollet

Felicita Urzi

Ola Magne Vagnildhaug

Evelien Van Roie

Hana Vankova

Kandy Velazquez

Hidetaka Wakabayashi

Tyrone Washington

Jan Waskowski

Debra Waters

Jie Wei

Steven Welc

Carly Welch

Christian Werner

Michael Wiggs

Simon Wing

Chang Won Won

Rob Wust

Elisabeth Wyart

Junjie Xiao

Chunyun Xiao

Xi Xiong

Kaiwen Xu

Yoshio Yamauchi

Dong Keon Yon

Mee‐Sup Yoon

Michael Yule

Nadège Zanou

Shu Zhang

Weihua Zhang

Qi Zhao

